# Secondary Inflammations in Chronic Renal Disease

**Published:** 1904-07-16

**Authors:** C. O. Hawthorne

**Affiliations:** Physician to the Central London Ophthalmic Hospital, Assistant Physician to the North-West London and Royal Waterloo Hospitals.


### HOSPITAL CLINICS.

### SECONDARY INFLAMMATIONS IN CHRONIC RENAL DISEASE.

A Clinical Lecture by C. O. HAWTHORNE, M.D., M.R.C.P., Physician to the Central London Ophthalmic Hospital, Assistant Physician to the North-West London and Royal Waterloo Hospitals.

THE cases on which this lecture is a commentary are not to be classed among the rarities of clinical experience. On the contrary, the disease from which the patients suffered is a very common one, and even the manner in which it here displayed itself is by no means very unusual. There are, however, certain clinical features of cases of this order which are apt to cause serious error both in diagnosis and prognosis, and hence the present examples may perhaps be studied with advantage. The first case is that of a man of 57 years, admitted to hospital with a complaint of severe pain in the region of the liver. The pain came on suddenly seven days before admission, and had apparently caused the patient great distress. Prior to this his history was that of a strong, healthy, temperate man. His general appearance was in entire harmony with this statement, and in his own view he was in perfect health, except for the pain in his upper right abdomen. Perhaps the first suggestion which occurs to the mind on hearing of the sudden development in a healthy adult of an acute pain in the region of the liver is that of gall-stone. All speculation in this and other directions was in the present instance rendered unnecessary by the detection over the extreme base of the right lung of undoubted evidences of pleural friction. At first sight, therefore, the announcement of pleurisy at the right base seemed both a confident and a sufficient diagnosis. The only feature of the case apparently calling for comment was the distribution of the pain and tenderness, not over the site of the pleurisy, but over the upper abdomen. Such an occurrence is presumably explained by the course of the lower intercostal nerves, and, in any event, it is not an uncommon clinical experience. Indeed, the first clinical lesson of the present case is to emphasise the necessity for remembering that abdominal pain may find its interpretation in an abnormal condition existing not below but above the diaphragm. Many illustrations of this truth have been recorded in the shape of mistaken diagnosis, and they all combine to enforce the oft-repeated claim for thoroughness and completeness in physical examination. This doctrine receives further emphasis from other aspects of the case now under review. It might, perhaps, not altogether unreasonably in the circumstances, have been concluded that a recognition of the existence of an acute pleurisy exhausted the diagnosis. An apparently healthy man in the prime of life is suddenly seized with acute pain, and physical examination detects pleural friction. Is there any necessity to attempt to go behind these facts? They form, it would seem, a definite and well-understood clinical picture. All the same, had the diagnosis halted at this point, a very grave error would have been committed. Now, what were the results of further examination? In the first place, it was observed that the temperature record was rather below than above the normal level—a somewhat startling fact in a case of acute pleurisy with severe and persisting pain. Again, examination of the urine showed a moderate quantity of albumen and the microscope revealed many hyaline and fatty tube casts. Hence it became necessary to consider whether or not the case included the existence of organic renal disease. No doubt, in many acute febrile processes—such for example as the specific fevers, pneumonia, etc.—albumen may appear in the urine without implying any permanent disease of the kidneys and without sensibly modifying the prognosis. It is generally assumed that such a condition depends on the febrile temperature and on the disturbances of which this is the expression. But in the present case there was no febrile temperature, and this in itself was sufficient to give pause to the conclusion that the albuminuria was a mere consequence of the pleurisy, and was of no serious moment. Further, whilst the so-called febrile albuminuria may be associated with the presence of tube casts, these are not usually so numerous as in the present case, and the existence of many casts showing fatty degeneration carries a strong suggestion of definite renal disease of a chronic order. And yet again, the urine was not sensibly diminished in quantity, it was pale in colour, and its specific gravity was less than 1,010—that is, its physical qualities were quite unlike the concentrated urine that usually attends the febrile process. Thus the absence of fever, the general characters of the urine, and the detection of numerous fatty casts were facts difficult to reconcile with the view that the albuminuria was a mere symptomatic expression of the general disturbance caused by an acute pleurisy. Nor was this all. Examination of the precordial region readily detected the signs of considerable hypertrophy of the left ventricle. The cardiac impulse was felt in the sixth intercostal space, and fully two inches to the outer side of the vertical line of the nipple. The force of the impulse and the vigour of the sounds at once negatived the view that this could be due to an acute dilatation caused by the severe pain or other constitutional disturbance from which the patient was suffering. The condition was certainly one of hypertrophy, and the absence of any murmur accompanying either of the sounds excluded the possibility that the cause of this could be disease of one or other of the cardiac valves. Such facts as visible pulsation and undue rigidity of the radial and other arteries, the existence of a pulse of sustained tension, and the marked accentuation of the aortic second sound afforded the necessary explanation, and showed the cardiac hypertrophy to be associated with a general condition of arterial degeneration and of abnormal tension in the arterial system. But, as is well known, these changes in the circulatory apparatus are among the generally recognised associations of one form of kidney disease, namely, chronic interstitial nephritis. Hence the conclusion suggested by the examination of the urine was placed altogether beyond question by the results obtained by the examination of the cardio-vascular system. The facts obtained from these two sources rendered it inevitable that in the diagnosis of the case chronic interstitial nephritis must of necessity be included. Had it been possible to have examined the fudus oculi, still further confirmation might have] been obtained in the shape of neuritis, retinitis, or vascular changes. But the patient was so restless in consequence of the pain that the ophthalmoscope could not be successfully employed; and, in view of the above facts, the diagnosis already suggested could not possibly be avoided.

But this is a position far removed from that presented by the facts of the case which first came under observation. These, considered alone, offered merely a diagnosis of acute pleurisy, and on the whole they accommodated themselves so satisfactorily to that diagnosis that there was some temptation to leave the case as an ordinary example of that disease. But a more responsible and exhaustive collection of facts by introducing conclusive evidence of the presence of organic renal disease showed the necessity for a considerable advance from such a position. Such a process avoided—and none other would have avoided—a grave error in diagnosis, and this, had it been committed, would have involved an equally serious mistake in prognosis. For whatever anxieties may be attached to a case of so-called "simple" acute pleurisy, they are as nothing compared to those which are suggested by the development of an acute pleurisy in the course of a case of chronic nephritis. The pleurisy in such circumstances is in all probability an expression of defect in the excretory power of the kidney, and results from the retention of waste products in the blood; and the prognosis in all such conditions is a most grave one. It is thus written in conspicuous characters across the record of the case that to secure an accurate diagnosis and an informed prognosis a complete examination is essential, and this even when a less extensive investigation affords what may be regarded as a sufficient explanation of the symptoms. Next appears the conclusion that a patient with chronic interstitial nephritis may reach an advanced stage of the disease without any symptoms causing him anxiety or even annoyance. The first active manifestation of this disease may, as the present record shows, be an acute pleurisy, and this it is to be specially noted may develop without any appreciable elevation of the temperature. It is, indeed, not too much to say that an acute pleurisy occurring in a patient who has reached middle life is an event which in itself ought to excite a suspicion of kidney disease, and the suspicion is all the more keen if there is at the same time no thermometric evidence of fever. Exactly the same remark may be made of pericarditis, and here more particularly there may be, not only absence of fever, but also absence of pain. This is illustrated in a second patient—also a man of 57 years—admitted to hospital, shortly after the case above described, with a complaint of breathlessness and general weakness. Albuminuria and tube-casts were detected on examination of the urine, and during patient's residence he developed the physical signs of consolidation of the upper lobe of the right lung, with blood-tinted sputa, exactly as these facts appear in an acute pneumonia. About the same time coarse pericardial friction was heard. But in spite of these complications the temperature never exceeded the normal, and the patient made no complaint of pain or other discomfort capable of being referred to the presence either of the pneumonia or the pericarditis. This "quiet" character of the secondary inflammations consequent on kidney disease is an important fact in the natural history of such disease, as unless it is remembered the occurrence of these developments may be overlooked, and thus their prognostic significance be lost. Their appearance, unfortunately, is a sign of bad omen. They are, only too frequently, terminal events in the history of renal disease, and to mistake them for "simple" inflammations, and to speak of them in terms based on such a view, is more than likely to receive a disastrous correction. The cases now reviewed illustrate this proposition, for the first patient died on the seventh and the latter on the sixteenth day after admission to hospital. Treatment directed to promote the excretory action of the kidneys, skin, and intestines proved, as it too often does in such circumstances, of no avail. There is, however, an aspect of this group of cases which is not without its therapeutic lesson. Allowing that the secondary inflammations which characterise them are excited by effete products which the kidneys have failed to remove from the blood it is still possible that other influences have some share in their production. Certain it is that, however produced, inflammations of mucous and serous membranes are disastrous events to any patient the subject of chronic renal disease. What, for example, in a healthy man would be a simple bronchial catarrh with a prompt resolution under some mild and simple treatment may readily, in a patient with a damaged kidney, be the immediate prelude to a fatal issue. Hence an important element in the treatment of the victim of chronic kidney disease is to secure his protection against circumstances which tend to determine these, for him, disastrous complications. This no doubt involves attention to the activity of the organs engaged in elimination. It is, indeed, in this direction much more than in attempts to reduce the loss of albumen —an ambition probably beyond the resources of art —that the pathway of safety is to be found. At the same time it is reasonable to believe that those ill-defined influences which are covered by the term "chill" may in the chronic albuminuric, as in the healthy—probably, indeed, more readily than in the healthy—cause pleurisy or bronchial catarrh or other similar events. It thus becomes a duty to warn such patients against these influences and to indicate suitable measure of climate, clothing, and general hygiene by which protection may be obtained. The two cases here reviewed show with painful distinctness how subtle, how serious, and how largely beyond therapeutic control are the various inflammatory developments which may occur in the course of chronic renal disease. They also enforce the duty of so guiding those who are the victims of this disease that the risks to which such patients are in a special degree liable may be reduced to the lowest level that circumstances permit.

